# Adding a C-terminal Cysteine (CTC) Can Enhance the Bactericidal Activity of Three Different Antimicrobial Peptides

**DOI:** 10.3389/fmicb.2018.01440

**Published:** 2018-06-28

**Authors:** Heng-Li Chen, Pei-Yi Su, Shu-Chen Kuo, Tsai-Ling Y. Lauderdale, Chiaho Shih

**Affiliations:** ^1^Institute of Biomedical Sciences, Academia Sinica, Taipei, Taiwan; ^2^National Institute of Infectious Diseases and Vaccinology, National Health Research Institutes, Zhunan, Taiwan

**Keywords:** antimicrobial peptide, drug resistance, *A. baumannii*, *S. aureus*, sepsis, lung infection

## Abstract

The emergence of antibiotic-resistant bacteria has threatened our health worldwide. There is an urgent need for novel antibiotics. Previously, we identified a novel 37-mer antimicrobial peptide (AMP), HBcARD, with broad spectrum antimicrobial activity. Here, we improved the efficacy of HBcARD, by re-engineering the peptide, including the addition of a new cysteine to its C-terminus (CTC). The new 28-mer derivative, D-150-177C, contains all D-form arginines, in addition to a C-terminal cycteine. This peptide can kill antibiotic-resistant clinical isolates of Gram-negative bacteria, and is more potent than the parental HBcARD peptide in a mouse sepsis model. In another lung infection mouse model, D-150-177C showed protection efficacy against colistin-resistant *Acinetobacter baumannii*. Unlike colistin, we observed no acute toxicity of D-150-177C *in vivo*. Interestingly, we found that CTC modification could enhance the antibacterial activity of several other AMPs, such as buforinII and lysin. The potential application and mechanism of this CTC method as a general approach to improving drug efficacy, warrants further investigation in the future.

## Introduction

Antibiotics have been used for the treatment of bacterial infection for more than 60 years. Over the past few decades, the extensive use of antibiotics has caused the rapid increase of drug resistance in both Gram-negative and Gram-positive bacteria. There are several life-threatening antibiotics-“ESKAPE" pathogens, which caused the majority of nosocomial infection, including *Enterococcus faecium*, *Staphylococcus aureus*, *Klebsiella pneumoniae*, *Acinetobacter baumannii*, *Pseudomonas aeruginosa*, and *Enterobacter* species (Rice, 2008). This situation was headlined as “Bad bugs, No drugs" by The Infectious Diseases Society of America (Boucher et al., 2009). It is therefore urgent to develop new antibiotics for clinical treatment.

Antimicrobial peptides (AMPs) play an important role in the host defense against pathogenic microbes (Zasloff, 2002; Mansour et al., 2014). To date, more than 2,500 natural AMPs have been discovered from various species, such as fungi, plants, and animals (Wang et al., 2016). One common feature of many AMPs, including melittin, defensin and magainin, is to adopt an amphipathic structure on the membrane of microbes. New derivatives with improved potency have largely been based on the structure-activity relationship, by using these natural AMPs as a reference template (Zelezetsky and Tossi, 2006; Fjell et al., 2012). Chimeric AMPs, fused from two different AMPs, have also been shown to improve the antimicrobial activity (Fox et al., 2012; Joshi et al., 2012; Ji et al., 2017). Other successful examples of AMP modification include substitutions with D-form amino acids, β-naphthylalanine, α,α-dialkyl amino acids and peptoids (Taira et al., 2010; Yu et al., 2011; Mojsoska et al., 2015; Chen et al., 2016; Khara et al., 2016). In summary, AMPs could be the next-generation antibiotics to overcome the problem of antibiotic-resistance (Hancock and Sahl, 2006; Nguyen et al., 2011; Afacan et al., 2012; Omardien et al., 2016; Ageitos et al., 2017).

We previously identified a novel AMP from human hepatitis B virus core (HBc) protein arginine-rich domain (ARD). This HBcARD peptide exhibited a broad spectrum antimicrobial activity against both Gram-negative and Gram-positive bacteria (Chen et al., 2013). *A. baumannii* is one of the most common nosocomial infection worldwide (Dijkshoorn et al., 2007; Kuo et al., 2012). Colistin and polymyxin B have been considered as the last hope antibiotics against Gram negative bacteria (Cai et al., 2015). We demonstrated previously that the HBcARD peptide can kill four out of four colistin-resistant *A. baumannii in vitro* (Chen et al., 2013). A lung infection mouse model by *A. baumannii* was established previously (Yang et al., 2016). It remains to be tested whether HBcARD peptide can show any protection efficacy in the mouse model infected with colistin-resistant *A. baumannii*.

To further improve the potency of our HBcARD AMP, we designed in this study a series of shorter derivative modified from the parental 37-mer HBcARD. By adding a cysteine at the carboxyl terminus (CTC) of a 27-mer peptide D-150-176, we designed a novel peptide D-150-177C, which can protect mice from death, when infected with either Gram-positive *S. aureus* or Gram-negative *A. baumannii*. This novel CTC modification strategy was also able to improve the antibacterial activities of other AMPs, such as buforin and lysin. The potential mechanism of efficacy enhancement by the CTC modification warrants further investigation.

## Materials and Methods

### Ethics Statement

This study was carried out in accordance with the recommendations stated in the Guide for the Care and Use of Laboratory Animals, National Research Council, 1996. All animal experiments were conducted under protocols approved by Academia Sinica Institutional Animal Care & Utilization Committee (ASIACUC permit number 12-02-322) and Institutional Animal Care and Use Committee of National Health Research Institutes (NHRI-IACUC-104139).

### Bacterial Isolates

The antimicrobial activities of HBcARD peptides were tested using a number of bacterial strains, including *Pseudomonas aeruginosa* Migula strains (ATCC 27853, and ATCC 9027), *Klebsiella pneumoniae* strain (ATCC 13884), *Escherichia coli strain* (ATCC 25922), *Staphylococcus aureus* strains (ATCC 25923, ATCC 29213, and ATCC 19636), and *Acinetobacter baumannii* strains (ATCC 17978, ATCC19606, TCGH 45530, and TCGH 46709). Colistin-resistant clinical isolates TCGH 45530 and TCGH 46709 were obtained from Tzu-Chi Buddhist General Hospital (TCGH) in Taiwan. Twenty drug-resistant, Gram-negative, clinical isolates, including *E. coli, K. pneumoniae, A. baumannii*, and *P. aeruginosa*, were obtained from Taiwan Surveillance of Antimicrobial Resistance program (TSAR), National Health Research Institutes (NHRI), Taiwan.

### Antimicrobial Assay

All peptides were purchased from Yao-Hong Biotechnology Inc. (Taipei, Taiwan). The minimum bactericidal concentration (MBC) was determined as described elsewhere (Chen et al., 2016). Briefly, bacteria were grown in Mueller-Hinton (MH) broth (Difco) to the mid-logarithmic phase at 37°C, and were diluted to 10^6^ CFU (colony formation unit)/ml in phosphate buffer (10 mM sodium phosphate and 50 mM sodium chloride, pH 7.2). Peptides were diluted in the same buffer. Fifty micro-liters of bacteria were mixed with 50 μl of serially diluted peptides, followed by incubation at 37°C for 3 h without shaking. At the end of incubation, bacteria were plated on MH agar. After incubation overnight at 37°C, the lowest peptide concentration that displayed no bacterial growth (zero colony) was determined as MBC. All peptides were tested in triplicate. For determining the minimum inhibitory concentration (MIC), bacteria were diluted to 10^6^ CFU/ml in MH broth. Peptides were also diluted in MH broth. The bacteria (50 μl) were incubated with serially diluted peptides (50 μl) at 37°C overnight with shaking. Growth of bacteria was measured by the optical density at 600 nm. The lowest peptide concentration, which showed the same OD600 value as the no peptide control, was defined as MIC. All peptides were tested in duplicate.

### *In Vivo* Animal Studies

Specific pathogen free (SPF) mice were housed in the individually ventilated cage (IVC) with light controlled in 12-h day-night periods and temperature controlled at 25°C. Each cage contains 5 mice. They were given standard laboratory food and water *ad libitum*. The sample size of animal experiments was estimated by the “resource equation” method (Charan and Kantharia, 2013).

We determined the *in vivo* protection efficacy of peptides in two different mouse infection models. In the mouse sepsis model (Chen et al., 2016), 3-week-old male ICR mice (∼20 g) were purchased from BioLASCO (Taiwan). These mice were inoculated intraperitoneally with *S. aureus* ATCC 19636 (4 × 10^6^ CFU/mouse). At 2 h post-inoculation, the mice were randomly separated into 5 groups and received peptides (5 or 10 mg/kg) or the PBS control, respectively. Each group contained 5 mice. Mortality was monitored daily for 7 days following the bacterial inoculation. This experiment was repeated twice. In the mouse lung infection model (Yang et al., 2016), 6–8-week-old male C57BL/6JNarl mice (∼27 g) were purchased from National Laboratory Animal Center, Taiwan. The inoculums (3.5 × 10^8^ CFU/mouse) were prepared by 1:1 mixing of LB culture and 10% mucin (Sigma). All mice were randomly separated into 3 groups and anesthetized by Isoflurane via inhalation, and inoculated intra-tracheally with colistin-resistant *A. baumannii* TCGH 46709. Two hours after inoculation, the mice were intraperitoneally injected daily with either colistin (5 mg/kg) (Fishbain and Peleg, 2010) or HBcARD derivative peptides (5 or 10 mg/kg), respectively, for three consecutive days. Each group contained 8 mice. Mortality was monitored every 12 h following the bacterial inoculation. This experiment was repeated twice.

To measure *in vivo* the acute toxicity, 3-week-old male ICR mice (∼20 g) were purchased from BioLASCO (Taiwan). The mice were randomly separated into 6 groups, and were intraperitoneally injected with D-150-177C at concentrations of 20, 40, 60, and 80 mg/kg. A last-line antibiotic, polymyxin B (20 and 50 mg/kg), was used as a control. Each group contained 5 mice. At 1 day post-injection, blood sample was collected from each mouse. The liver function of mice was determined by measuring the ALT level in the blood. The survival rates of injected mice were monitored for 7 days.

### Statistical Analysis

Statistical analysis was performed using the Graphpad software. Survival curves of all groups were plotted by the Kaplan–Meier method and analyzed by the log-rank test.

## Results

### The Importance of the Terminal Cysteine

**FIGURE 1:**
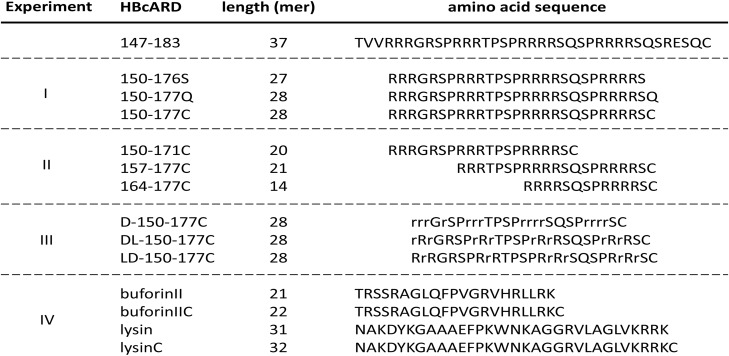
Amino acid sequences of HBcARD peptides tested for bactericidal activity in this study. HBcARD 147-183 is the prototype peptide. The derivative peptides were synthesized for optimization of bactericidal activity in this study. Uppercase letters indicate L-form amino acids. Lowercase letters indicate D-form amino acids. For experiment-I, peptides with different C-terminal amino acid compositions (150-176S, 150-177Q, and 150-177C) were compared for the antibacterial activity *in vitro* and *in vivo*. For experiment-II, N-terminus- and C-terminus-truncated HBcARD peptides (150-171C, 157-177C, and 164-177C) were generated. These peptides contain different arginine contents. For experiment-III, peptides containing all-D-arginine or partial-D-arginine substitution (D-150-177C, DL-150-177C, and LD-150-177C) were compared for their respective bactericidal activity. For experiment-IV, peptides (buforinII, buforinIIC, lysin and lysinC) were compared for the enhancement of *in vitro* antibacterial activity by the CTC method.

In our previous results (Chen et al., 2013), we found that the antimicrobial activity against *S. aureus* was diminished, when the HBcARD peptide lost the last 8 amino acids (SQSRESQC). To further improve the potency of our lead compound HBcARD, we tested the antimicrobial activity of several HBcARD derivatives, including peptides modified by truncation and D-arginine substitution for L-arginine (**Figure 1**). The antimicrobial activities of these peptides were determined by minimal bactericidal concentration (MBC). Like the parental peptide HBcARD 147-175 (Chen et al., 2013), the derivative peptides HBcARD 150-176S and HBcARD 150-177Q showed no detectable bactericidal activity against *S. aureus* (**Table 1**). To mimic the parental peptide HBcARD 147-183 with a cysteine at the carboxyl terminus, we designed another derivative HBcARD 150-177C, by replacing the terminal Q (glutamine) residue of HBcARD 150-177Q with a C (cysteine) residue (**Figure 1**). The results showed that this Q-to-C substitution effectively rescued the bactericidal activity against three *S. aureus*. In addition, HBcARD 150-177C also retained the same spectrum and potency of antimicrobial activity against a number of Gram-negative bacteria (**Table 1**). We asked next whether the length of HBcARD 150-177C peptide (28-mer) can be further reduced. The potency against *S. aureus* ATCC19636 was no longer detectable up to 57.2 mg/L for peptides 150-171C (20-mer), 157-177C (21-mer), and 164-177C (14-mer). Altogether, these results here indicated that both arginine and C-terminal cysteine are important for antibacterial activity.

**Table 1:** Minimum bactericidal concentration (MBC) of HBcARD peptides against various Gram-negative and Gram-positive bacteria.

Bacteria strains	MBC (mg/L)						
	147-183	150-176S	150-177C	150-177Q	150-171C	157-177C	164-177C
**Gram-positive**						
*S. aureus* ATCC19636	18.3	ND	7.1	ND	ND	ND	ND
*S. aureus* ATCC25923	18.3	ND	7.1	ND	–	–	–
*S. aureus* ATCC29213	18.3	ND	7.1	ND	–	–	–
**Gram-negative**						
*P. aeruginosa* ATCC9027	9.2	–	7.1	–	–	–	–
*P. aeruginosa* ATCC27853	9.2	–	7.1	–	–	–	–
*E. coli* ATCC25922	18.3	–	7.1	–	–	–	–
*A. baumannii* ATCC17978	2.3	–	1.8	–	–	–	–
*A. baumannii* ATCC19606	2.3	–	0.9	–	–	–	–
*A. baumannii* ATCC45530	2.3	–	1.8	–	–	–	–
*A. baumannii* ATCC46709	4.6	–	3.6	–	–	–	–

*ND, not detectable at the concentration up to 57.2 mg/L; –, not determined.*

### Protection Efficacy of Modified HBcARD in Two Animal Models

**FIGURE 2:**
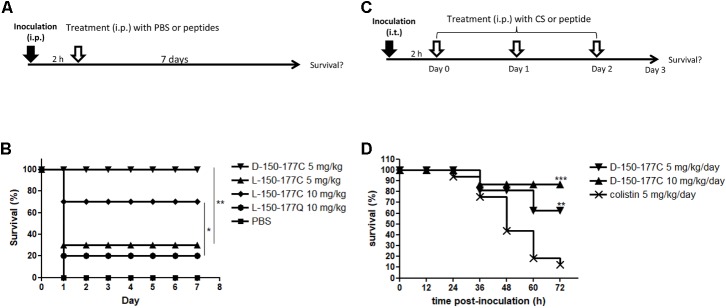
Comparisons of *in vivo* protection efficacy of HBcARD derivatives in two animal models. **(A)** In a mouse sepsis model, ICR mice (*n* = 10/group) were i.p. inoculated with *S. aureus* ATCC19636 (4^∗^10^6^/mouse) before treatments at 2 h post-inoculation. The survival rates were monitored for 7 days after injection. **(B)** All PBS control mice died at day 1, and only 20% of mice treated with a peptide L-150-177Q survived. Administration of L-150-177C peptide at the dose of 5 and 10 mg/kg can rescue 30 and 70% of mice from death, respectively. When treated with 5 mg/kg of D-150-177C, all mice survived. **(C)** In a lung infection mouse model, 6–8-week-old male C57BL/6JNarl mice were anesthetized by Isoflurane via inhalation, before intra-tracheal (i.t.) inoculation with *A. baumannii* (3.5 × 10^8^ CFU/mouse). Two hours after inoculation, mice were intraperitoneally (i.p.) injected daily with either colistin (CS) or HBcARD peptides for three consecutive days. The survival rates were monitored for 3 days after infection. **(D)** C57BL/6JNarl mice (16 mice/group) were i.t. inoculated with *A. baumannii* TCGH46709 (colistin-resistant strain) before treatment with either colistin (5 mg/kg) or D-150-177C peptide (5 and 10 mg/kg). There was a dose-dependent effect on the survival rate of mice receiving D-150-177C. The survival rates of mice treated with D-150-177C were significantly higher than that with colistin treatment. This result indicated that D-150-177C was more potent than colistin for killing colistin-resistant *A. baumannii*. ^∗^*P* < 0.05; ^∗∗^*P* < 0.01; ^∗∗∗^*P* < 0.001.

As shown in **Figure 2A**, we compared the *in vivo* protection efficacies between HBcARD 150-177Q and 150-177C peptides in the ICR mouse sepsis model. The mice (∼20 g) were i.p. (intraperitoneally) inoculated with *S. aureus* ATCC19636 (4 × 10^6^ CFU/mouse), followed by treatments with peptides or the PBS control at 2 h post-inoculation. We monitored the survival rate for 7 days (**Figure 2B**). All mice treated with PBS (10/10) died at day 1 post-inoculation. Consistent with its low *in vitro* MBC against *S. aureus*, administration of L-150-177Q peptide at the dose of 10 mg/kg showed a very low protection efficacy (2/10 survival). In contrast, administration of L-150-177C peptide at the same dose protected 70% of mice (7/10) from death (*p* < 0.05). It appears that the C-terminal cysteine is very critical for the *in vivo* protection as well. Next, we compared the *in vivo* protection efficacy of HBcARD 150-177C peptides containing either D-form or L-form arginines in this same model. The protection efficacy of peptide L-150-177C was 30% (3/10) at 5 mg/kg, whereas D-150-177C can protect all mice (10/10) from death at the same dose (**Figure 2B**). The results indicated that D-150-177C had a stronger protection effect than L-150-177C (*p* < 0.01).

In addition to the Gram-positive *S. aureus*, we performed another mouse lung infection model to investigate the protection of D-150-177C peptide against the Gram-negative *A. baumannii* (**Figure 2C**). C57BL/6JNarl mice (∼27 g) were anesthetized and intra-tracheally inoculated with *A. baumannii* (3.5 × 10^8^ CFU/mouse). At 2 h post-inoculation, the mice received i.p. injection of either D-150-177C or colistin, respectively, for three consecutive days (1 injection per day). In one experiment with colistin-sensitive *A. baumannii* ATCC17978 strain, the administration of D-150-177C resulted in a survival rate comparable to that of colistin treatment (data not shown). In the other experiments infected with colistin-resistant *A. baumannii* TCGH 46709 strain, 12.5% mice (2/16) were poorly rescued by colistin (5 mg/kg/day). In contrast, peptide D-150-177C at the dose of 5 and 10 mg/kg/day protected 62.5% (10/16, *p* < 0.01) and 87.5% (14/16, *p* < 0.001) of mice from death, respectively (**Figure 2D**). These results indicated that D-150-177C exhibited a better protection efficacy than colistin for the colistin-resistant *A. baumannii.*

### Peptide D-150-177C Showed Very Low *in Vivo* Toxicity Than Polymyxin B

**FIGURE 3:**
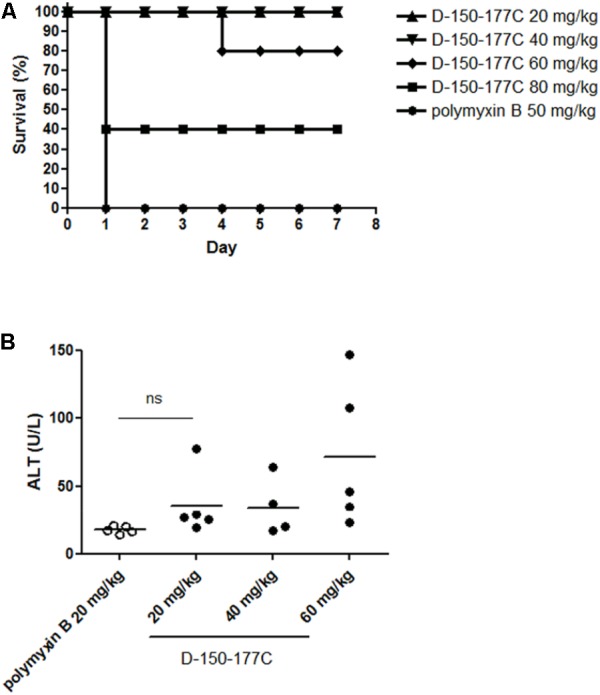
*In vivo* toxicity of D-150-177C peptide. At day 0, male ICR mice (5 mice/group) were i.p. injected with different doses of D-150-177C peptide (20–80 mg/kg) and polymyxin B (50 mg/kg), respectively. **(A)** The survival rates of all groups were monitored for 7 days. All mice treated with polymyxin B (50 mg/kg) died at day 1. In contrast, no acute toxicity was observed in mice treated with 20 and 40 mg/kg D-150-177C. When we increased the dose to 60 and 80 mg/kg, the survival rates of mice were decreased to 80 and 40%. **(B)** Alanine aminotransferase activity (ALT) was measured in serum samples of mice treated with D-150-177C or polymyxin B at day 1. The horizontal bars represent the means of ALT values of ICR mice in different experimental groups. Treatment with D-150-177C in the dose of 20 and 40 mg/kg showed a slight increase in the ALT level than treatment with polymyxin B at 20 mg/kg. The ALT level of mice treated with 60 mg/kg D-150-177C was significantly higher than that treated with polymyxin B at 20 mg/kg (*P* < 0.01).

Polymyxins are the last-line antibiotics, but could be toxic at higher dose. We compared the acute toxicity of peptide D-150-177C with polymyxin B using ICR mice. The mice (∼20 g) were i.p. injected with D-150-177C peptide (20–80 mg/kg body weight). Polymyxin B (50 mg/kg body weight) was used as a control antibiotic. As shown in **Figure 3A**, all mice treated with peptide D-150-177C at the dose of 20 and 40 mg/kg survived; while at 60 and 80 mg/kg, the survival rates dropped from 100 to 80% (4/5) and 40% (2/5). The LD_50_ of D-150-177C peptide was estimated to be 75 mg/kg. In contrast, all mice died of polymyxin B at 50 mg/kg. Therefore, the acute toxicity of peptide D-150-177C is significantly lower than polymyx B. On the other hand, we also monitored liver injury by measuring serum ALT levels in blood samples from mice at 1 day post-injection. At the dose of 60 mg/kg, D-150-177C peptide showed slightly elevated level of ALT compared to mice treated with 20 mg/kg polymyxin B (**Figure 3B**).

### Antimicrobial Activity of Peptide D-150-177C Against Clinical Isolates

We determined the antimicrobial activity of peptide D-150-177C against 20 clinical isolates obtained from TSAR (Taiwan Surveillance of Antimicrobial Resistance), including *E. coli, K. pneumoniae, A. baumannii*, and *P. aeruginosa*. As shown in **Table 2**, 19 out of 20 isolates were drug-resistant to various antibiotics. Despite their diverse drug-resistant phenotypes, they were invariably inhibited by the peptide D150-177C at the range of 16 to 32 mg/L except for *K. pneumoniae.*

**Table 2:** Minimum inhibitory concentration (MIC) of D-150-177C against antibiotics-resistant bacterial isolates.

Clinical isolates		Origin	MIC (mg/L)	Resistance phenotype
*E. coli*	1	Liver pus	16	AMP, PIP, SXT
	2	Urine	16	AMP, CFZ-UTI, CIP, FEP, FRX, FTX, GEN, LEV
	3	Urine	16	AMP, ATM, CFZ, CIP, FEP, FRX, FTX, LEV, SXT, TZP
	4	Urine	16	AMK, AMP, ATM, CAZ, CFZ, CIP, FEP, FOX, FRX, FTX, GEN, LEV, SXT
	5	Urine	16	AMP, CAZ, CFZ, COL, FOX, FRX, FTX, SXT
*K. pneumoniae*	1	Liver abscess	>32	AMP
	2	Urine	>32	AMP, CIP, COL, LEV, SXT
	3	liver	32	AMP
	4	Urine	>32	AMP, ATM, CAZ, CFZ, CIP, FEP, FOX, FRX, FTX, LEV, TZP
	5	Liver abscess	>32	AMP
*A. baumannii*	1	Urine	32	AMK, CAZ, CIP, DOR, FTX, GEN, IMP, LEV, MEM, PIP, TZP
	2	Urine	32	AMK, CAZ, CIP, DOR, FEP, FTX, GEN, IMP, LEV, MEM, PIP
	3	Urine	32	AMK, AMS, CAZ, CIP, DOR, FEP, FTX, GEN, IMP, MEM, PIP, TZP
	4	Urine	16	CAZ, CIP, DOR, FTX, GEN, IMP, LEV, MEM, PIP, TZP
	5	Urine	16	–
*P. aeruginosa*	1	Urine	16	CAZ, CIP, FEP, GEN, IMP, LEV, MEM
	2	Urine	32	ATM, CIP, DOR, IMP, LEV, MEM
	3	Urine	16	ATM, CIP, IMP, LEV, MEM
	4	Urine	32	AMK, CIP, DOR, FEP, GEN, IMP, LEV, MEM, PIP, TZP
	5	Urine	32	ATM, CAZ, IMP, MEM, PIP, TZP

*AMK, Amikacin; AMP, Ampicillin; AMS, Ampicillin/Sulbactam; ATM, Aztreonam; CAZ, Ceftazidime; CFZ, Cefazolin (based on interpretive criteria for uncomplicated urinary tract infection); CIP, Ciprofloxacin; COL, Colistin; DOR, Doripenem; EPT, Ertapenem; FEP, Cefepime; FOX, Cefoxitin; FRX, Cefuroxime; FTX, Cefotaxime; GEN, Gentamicin; IMP, Imipenem; MEM, Meropenem; PIP, Piperacillin; SXT, Trimethoprim/sulfamethoxazole; TZP, Piperacillin/Tazobactam.*

### Comparison of *in Vivo* Protection Efficacies Among Various Modified 150-177C Peptides

**FIGURE 4:**
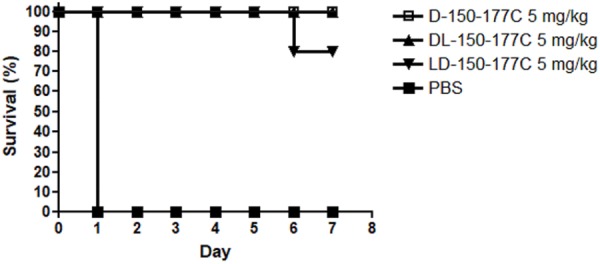
*In vivo* protection efficacy of partially modified 150-177C peptides in a mouse sepsis model. Two hours post-inoculation, the mice (*n* = 10/group) were treated with 5 mg/kg of HBcARD peptides modified with 50 and 100% D-arginine substitutions (D-150-177C, DL-150-177C, and LD-150-177C in **Figure 1**). All of these peptides can successfully protect *S. aureus*-infected mice from death with a high efficiency (80 – 100%).

Peptide D-150-177C contains a total of 14 L-arginines substituted with 14 D-arginines (**Figure 1**). This modified peptide showed increased antimicrobial activity (**Figure 2**). Because D-arginine is far more expensive than L-arginine, we investigated the possibility of partial D-arginine substitution in the mouse sepsis model. The *in vivo* protection efficacies were compared between peptides containing complete or partial D-arginine substitutions. We designed two partial D-arginine-substituted peptides, designated as DL- and LD-150-177C (**Figure 1**). Both peptide DL-150-177C and all-D-arginine peptide D-150-177C protected all mice from death (**Figure 4**). While peptide LD-150-177C appeared to be less protective (80% survival rate), but there were no significant difference between these two (*P* > 0.05).

### Enhancement of Antimicrobial Activity via a C-terminal Cysteine (CTC)

To determine whether the CTC modification strategy can be applied to enhance the spectrum and efficacy of other AMPs, we compared the antibacterial activities between AMPs with and without the CTC modification. We chose buforinII (Park et al., 1998) and lysin (Thandar et al., 2016), whose amino acid sequences bear no resemblance to HBcARD (**Figure 1**). As shown in **Table 3**, buforinIIC exhibited fourfold enhancement over buforinII in the antimicrobial activity against *P. aeruginosa*. This modification also enhanced the antimicrobial activity against both *K. pneumoniae* and *A. baumannii*, albeit it showed no effect against *S. aureus* at the concentration up to 1024 mg/L. Similar enhancement of antimicrobial activity by CTC modification was observed in another AMP lysin. Remarkably, the MBC against *P. aeruginosa*, *K. pneumoniae, A. baumannii*, and *S. aureus* were all significantly enhanced. For example, compared to its parental lysin without a C-terminal cysteine, LysinC exhibited a 4-fold, >8-fold, >8-fold, and 32-fold decrease in MBC against *P. aeruginosa, K. pneumonia, S. aureus*, and *A. baumannii*, respectively.

**Table 3:** Enhancement of antimicrobial activity of buforin and lysin.

Bacteria	MBC (mg/L)			
	BuforinII	BuforinIIC	Lysin	LysinC
*P. aeruginosa* ATCC9027	16	4	64	16
*K. pneumoniae* ATCC13884	ND	>1024	ND	128
*A. baumannii* ATCC17978	>1024	1024	>1024	32
*S. aureus* ATCC 19636	ND	ND	ND	128

*ND, not detectable at the concentration up to 1024 mg/L.*

## Discussion

Hydrophobic residues in the amphipathic structure of AMPs are known to play an important role in the structure-activity studies (Fjell et al., 2012). However, in this study, we observed similar antimicrobial activity of HBcARD after deleting three hydrophobic residues (TVV) from the N-terminus (**Figure 1** and **Table 1**). It suggests that these hydrophobic residues at the N-terminus were not required for the antimicrobial activity. At the C-terminus of HBcARD147-183, the last 8 amino acids (SQSRESQC) appeared to be critical for the potency against *S. aureus* (Chen et al., 2013). It is unclear how these terminal residues could affect the potency. We tested the hypothesis whether the terminal cysteine residue in peptide 150-177C could contribute to the bactericidal activity, by comparing peptides 150-176S, 150-177Q, and 150-177C (**Figure 1**). Only 150-177C exhibited marked *in vitro* and *in vivo* activity against *S. aureus* (**Table 1** and **Figure 2B**). Therefore, the C-terminal cysteine residue (CTC) can enhance the antimicrobial activity of HBcARD peptide against *S. aureus*. In addition to the C-terminal cysteine, the arginine content appeared to be important as well, based on the deletion mapping experiment (peptides 150-171C, 157-177C, and 164-177C). In summary, a C-terminal cysteine and a sufficient number of arginine are important for the HBcARD peptide.

In our previous studies, we successfully improved the *in vivo* potency of the prototype 37-mer HBcARD by D-arginine substitution in a mouse sepsis model (Chen et al., 2016). Using this same strategy, we demonstrated here that a 28-mer peptide 150-177C significantly improved the protection efficacy from 30% by the L-150-177C to 100% by D-150-177C at the dose of 5 mg/kg (**Figure 2B**). So far, our most active derivative is the 28-mer D-150-177C peptide. To reduce the cost for D-amino acids in peptide synthesis, we engineered peptides DL-150-177C and LD-150-177C, which contain only approximately 50% of D-arginine substitution. These partially D-substituted peptides displayed bactericidal activity similar to peptide D-150-177C, which contains 100% of D-arginines. It is possible that the protection efficacy can be maintained or improved by further reduction in the number of D-amino acid replacement in the future. Moreover, in our current assay, we measured only the survival rate as the end point. It is worth mentioning here that in our previous studies, bacterial burdens in spleen, liver, and blood were reduced by approximately 100-fold in all tissues in mice treated with full-length HBcARD (Chen et al., 2013).

Drug-resistance bacteria not only can cause therapeutic complications, but also increase the economic burden in hospitals (Perencevich et al., 2003). Therefore, we determined the antimicrobial activity of D-150-177C against 19 clinical isolates with a wide spectrum of drug resistance profiles (**Table 2**). Except for most strains of *K. pneumonia*, peptide D-150-177C, in the concentration between 16 and 32 mg/L, inhibited these drug-resistant *E. coli*, *A. baumannii*, and *P. aeruginosa*. Because the MIC values between drug-sensitive and drug-resistant strains are similar (**Table 2** and data not shown), we observed no cross-resistance *in vitro* from these clinical isolates to D-150-177C.

Polymyxin B and colistin (polymyxin E) are the “last-line” antibiotic peptides currently used in clinical medicine for treatment of drug-resistant Gram-negative bacteria (Cai et al., 2015). However, many cases of colistin- and polymyxin B-resistant strains are emerging, worldwide (Fernandez et al., 2010; Cai et al., 2012; Baadani et al., 2013; Lesho et al., 2013; Lopez-Rojas et al., 2013). *A. baumannii* is one of the most common antibiotic-resistant pathogens globally, which can cause a wide variety of infections in lung, bloodstream, urinary tract and surgical wounds (Dijkshoorn et al., 2007; Kuo et al., 2012). In our mouse model i.t. infected with colistin-resistant *A. baumannii*, treatment with D-150-177C can protect 90% of mice from death (**Figure 2D**). The lack of cross-resistance *in vivo* suggested that colistin and D-150-177C must have different modes of action.

A high rate of nephrotoxicity is known to be associated with polymyxins (Doshi et al., 2011; Abdelraouf et al., 2012; Omrani et al., 2015; Roberts et al., 2015). In our acute toxicity mouse model, 80% of mice receiving 60 mg/kg peptide D-150-177C survived, while 100% of mice died of polymyxin B at 50 mg/kg (**Figure 3A**). Although the mice receiving peptide D-150-177C (60 mg/kg) had an elevated ALT level compared to polymyxin B (20 mg/kg) treated mice (**Figure 3B**), the average ALT value appeared to fall in the range of the basal level (less than 100 IU/L) of untreated mice (see Materials and Methods). Taken together, treatment with D-150-177C peptide appeared to be well tolerated in the mouse model. Finally, in our current study, we performed no accumulative toxicity assays. This is because in our current short-term study, we tested only one-shot treatment for the sepsis model, and only three shots in 3 days for the lung infection model (**Figure 2**).

Ionic strength in the medium has been shown to influence the antibacterial activities of human beta-defensin 3 (hBD-3) derivatives (Boniotto et al., 2003; Kluver et al., 2005). In addition, it has been reported that polyanionic peptides present in LB and BHI media may form electrostatic complexes with cationic polymers, which would decrease the potency by diminishing the binding to the anionic lipopolysaccharide layer of *E. coli.* (Choi et al., 2014). In our studies, we noted that the MIC values were not always in parallel with the MBC values. For example, the MICs of D-150-177C for *A. baumannii* were 16–32 mg/L (**Table 2**), whereas its MBCs for *A. baumannii* were 1–4 mg/L (**Table 1**). Comparing to the D-150-177C peptide, colistin has a lower MIC around 4 mg/L for *A. baumannii* ATCC17978 strain. Despite their significant difference in the MIC values, we observed similar protection efficacies between colistin and D-150-177C in the animal model i.t. infected with *A. baumannii*. Therefore, the MIC values *in vitro* do not always reflect the *in vivo* antibacterial activity. Using the 0.5x MH broth, we found that the MIC values became closer to the MBC values (data not shown). Altogether, it is important to include an *in vivo* assay in an animal model, when the antibacterial activity is being evaluated, as the *in vitro* MIC assay could be subject to the influence of ionic strength in the broth.

Previously, we demonstrated that the C-terminal octamer peptide SQSRESQC of HBcARD is important for the antibacterial activity against *S. aureus* (Chen et al., 2013). This observation was confirmed recently. When phage lysin was conjugated with this octamer peptide of HBcARD or a scrambled peptide, the antibacterial activity of phage lysin was enhanced (Thandar et al., 2016). The principal determinant for this activity enhancement has remained ill-defined. In our investigation on the modification of HBcARD, we invented a CTC modification strategy (**Table 3**). In this approach, antibacterial activity or spectrum, including Gram-positive and Gram-negative, can be improved by adding an exogenous cysteine to the end of the C-terminus of an AMP, regardless of its sequence context. We cited here two such successful examples using the less potent buforinII and lysin. On the other hand, we experienced unsuccessful examples via CTC modifications, such as indolicidin, magainine and epinecidin-1 (data not shown).

It remains unclear what could be the mechanism for the CTC-mediated enhancement. It is unlikely that the effect is simply due to the peptide dimerization via the cysteine disulfide bond formation. The CTC enhancement effects are variable, depending on the bacteria being treated; however, the increased antibacterial activities are in general far greater than twofold. To date, a rich AMP database includes near three thousand naturally occurring AMPs (Wang et al., 2016). It should be rewarding to explore systematically, whether this simple CTC modification approach could help broaden the spectrum and boost the activity of these AMPs against various drug-resistant microorganisms in clinical medicine.

## Author Contributions

H-LC, S-CK, and CS designed the experiments. H-LC, S-CK, P-YS performed the experiments. All authors analyzed the data. CS, T-LL, and H-LC prepared the manuscript.

## Conflict of Interest Statement

The authors declare that the research was conducted in the absence of any commercial or financial relationships that could be construed as a potential conflict of interest.
